# Gut microbiota from patients with COVID-19 cause alterations in mice that resemble post-COVID symptoms

**DOI:** 10.1080/19490976.2023.2249146

**Published:** 2023-09-05

**Authors:** Viviani Mendes de Almeida, Daiane F. Engel, Mayra F. Ricci, Clênio Silva Cruz, Ícaro Santos Lopes, Daniele Almeida Alves, Mirna d’ Auriol, João Magalhães, Elayne C. Machado, Victor M. Rocha, Toniana G. Carvalho, Larisse S. B. Lacerda, Jordane C. Pimenta, Mariana Aganetti, Giuliana S. Zuccoli, Bradley J. Smith, Victor C. Carregari, Erika da Silva Rosa, Izabela Galvão, Geovanni Dantas Cassali, Cristiana C. Garcia, Mauro Martins Teixeira, Leiliane C. André, Fabiola Mara Ribeiro, Flaviano S. Martins, Rafael Simone Saia, Vivian Vasconcelos Costa, Daniel Martins-de-Souza, Philip M. Hansbro, João Trindade Marques, Eric R. G. R. Aguiar, Angélica T. Vieira

**Affiliations:** a Laboratory of Microbiota and Immunomodulation - Department of Biochemistry and Immunology, Institute of Biological Sciences, Universidade Federal de Minas Gerais - UFMG, Belo Horizonte, Brazil; b Department of Clinical Analysis, School of Pharmacy, Universidade Federal de Ouro Preto - UFOP, Ouro Preto, Brazil; c Laboratory of Virus Bioinformatics - Department of Biological Science, Center of Biotechnology and Genetics, Universidade Estadual de Santa Cruz - UESC, Ilhéus, Brazil; d Laboratory of RNA Interference and Antiviral Immunity - Department of Biochemistry and Immunology, Institute of Biological Sciences, Universidade Federal de Minas Gerais - UFMG, Belo Horizonte, Brazil; e Laboratory of Toxicology - Department of Clinical and Toxicological Analysis, Faculty of Pharmacy, Universidade Federal de Minas Gerais - UFMG, Belo Horizonte, Brazil; f Laboratory of Neurobiochemistry - Department of Biochemistry and Immunology, Institute of Biological Sciences, Universidade Federal de Minas Gerais - UFMG, Belo Horizonte, Brazil; g Center for Research and Development of Drugs - Department of Morphology, Institute of Biological Sciences, Universidade Federal de Minas Gerais - UFMG, Belo Horizonte, Brazil; h Laboratory of Neuroproteomics - Department of Biochemistry and Tissue Biology, Institute of Biology, Universidade do Estado de Campinas - UNICAMP, Campinas, Brazil; i Laboratory of Comparative Pathology - Department of Pathology, Universidade Federal de Minas Gerais - UFMG, Belo Horizonte, Brazil; j Laboratory of Respiratory Viruses and Measles, Instituto Oswaldo Cruz - Fiocruz, Rio de Janeiro, Brazil; k Laboratory of Biotherapeutic Agents - Department of Microbiology, Institute of Biological Sciences, Universidade Federal de Minas Gerais - UFMG, Belo Horizonte, Brazil; l Laboratory of Intestinal Physiology - Department of Physiology, Ribeirão Preto Medical School, Universidade de São Paulo, Ribeirão Preto, Brazil; m D’Or Institute for Research and Education, São Paulo, Brazil; n Experimental Medicine Research Cluster, Universidade do Estado de Campinas - UNICAMP, Campinas, Brazil; o National Institute of Biomarkers in Neuropsychiatry, National Council for Scientific and Technological Development, São Paulo, Brazil; p Centre for Inflammation, Centenary Institute and University of Technology Sydney, Sydney, Australia; q CNRS UPR9022, University of Strasbourg, Strasbourg, France

**Keywords:** COVID-19, SARS-CoV-2, post-COVID, microbiota, inflammation, antimicrobial-resistance

## Abstract

Long-term sequelae of coronavirus disease (COVID)-19 are frequent and of major concern. Severe acute respiratory syndrome coronavirus 2 (SARS-CoV-2) infection affects the host gut microbiota, which is linked to disease severity in patients with COVID-19. Here, we report that the gut microbiota of post-COVID subjects had a remarkable predominance of *Enterobacteriaceae* strains with an antibiotic-resistant phenotype compared to healthy controls. Additionally, short-chain fatty acid (SCFA) levels were reduced in feces. Fecal transplantation from post-COVID subjects to germ-free mice led to lung inflammation and worse outcomes during pulmonary infection by multidrug-resistant *Klebsiella pneumoniae*. transplanted mice also exhibited poor cognitive performance. Overall, we show prolonged impacts of SARS-CoV-2 infection on the gut microbiota that persist after subjects have cleared the virus. Together, these data demonstrate that the gut microbiota can directly contribute to post-COVID sequelae, suggesting that it may be a potential therapeutic target.

## Introduction

The newly emerged β-coronavirus, severe acute respiratory syndrome-coronavirus-2 (SARS-CoV-2), has caused more than 618 million confirmed cases and 6,8 million deaths globally as of March 2023.^1^ After the clearance of SARS-CoV-2, long-term complications of coronavirus disease-2019 (COVID-19) are common even among patients with mild or asymptomatic disease during the acute phase.^2–4^ SARS-CoV-2 requires the angiotensin-converting enzyme 2 (ACE2) and Transmembrane Serine Protease 2 (TMPRSS2) to infect lung cells.^5^ The gastrointestinal tract expresses high levels of these receptors and is also vulnerable.^6,7^ Gut microbiota has been shown to influence COVID-19 severity and long-term post-COVID effects.^7,8^

The human gut microbiome includes trillions of microorganisms, primarily bacteria, which form a complex and well-recognized ecosystem. An imbalance in the gut microbiota composition, referred to as dysbiosis, is a major factor in disease development and can be caused by viral infections and other respiratory challenges.^8–11^ Individuals who experience severe COVID-19 have reduced diversity and abundance of the commensal gut microbiota.^9,12,13^ Dysbiosis of the gut microbiota was observed up to 1 year after the initial infection and virus clearance post-COVID.^12^ Thus, these long-term changes in the gut microbiota could contribute to the symptoms of long-COVID, but there is currently no direct evidence for this link.^14,15^

Here, we investigated whether the gut microbiota derived from individuals previously infected with SARS-CoV-2, who had mild or no symptoms could induce post-COVID consequences. To investigate this, we transferred human fecal microbiota to germ-free mice as an experimental approach. Our data provide evidence that gut microbiota from COVID-19 patients can cause sequelae from infection in the absence of SARS-CoV-2, including the induction of lung inflammation and brain dysfunction.

## Results

### Comparison of clinical, dietary, and microbiological parameters between controls (non-COVID) and post-COVID subjects

**Figure 1.**
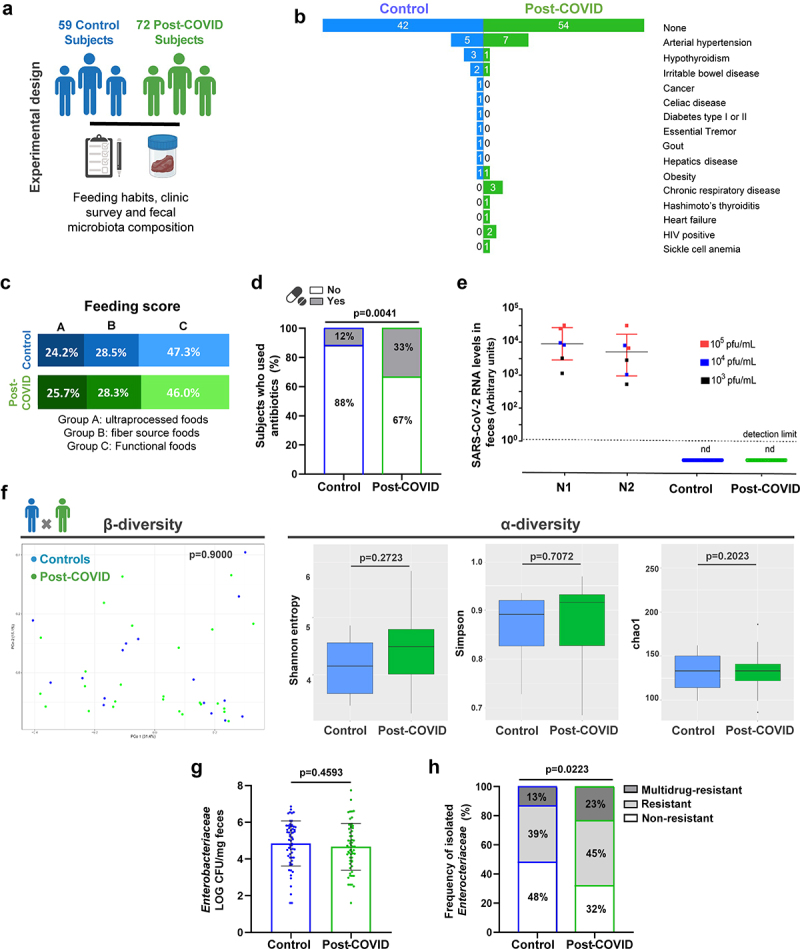
Clinical characteristics and food habits were associated with gut microbiota composition and an antimicrobial resistance profile in *Enterobacteriaceae* species of post-COVID and control human subjects. (a) experimental design: collection of feeding habits, clinic survey, and fecal microbiota composition analysis of 59 control and 72 post-COVID subjects (*N* = 131). (b) Co-morbidities in control and post-COVID groups. (c) feeding composition (*N* = 131). (d) antibiotic-treated control and post-COVID subjects (*N* = 131). (e) SARS-CoV-2 quantification by RT-qPCR in the feces of control and post-COVID subjects, a.U.: arbitrary units (*N* = 131). (f) 16S rRNA sequencing of gut microbiota from control and post-COVID subjects at the family level (*N* = 44). Principal Component analysis based on weighted Unifrac distances (*p* = 0.900), a β-diversity index (*N* = 44). α-diversity analysis based on Shannon, Simpson, and Chao1 indexes (*N* = 44). (g) *Enterobacteriaceae* quantification in fecal samples of the subjects (*N* = 131). (H) Frequency of *Enterobacteriaceae* strains present in the fecal samples of human subjects as multidrug-resistant, resistant, or nonresistance (*N* = 131). Statistical analysis: Fisher’s exact test was used in D, Wilcoxon and PerMANOVA pairwise tests were used in F, unpaired Student’s *t*-test was used in G, and Chi-square test was used in H. Data are shown as mean and standard deviation (SD). See also Figure S1.

We recruited 131 volunteers: seventy-two (55%) subjects had SARS-CoV-2 infection (post-COVID group), and fifty-nine (45%) subjects were COVID-19-naïve healthy control subjects (Figure 1a). The experimental design and clinical characteristics of all the subjects are summarized in Figure S1 and Table 1, respectively. Among the post-COVID subjects: sixty-four (89%) had mild/moderate illness during the symptomatic period, and feces were collected between 1 and 4 months after initial symptoms. The exact time for samples collected from infection was summarized in Supplementary Table S1. Additionally, thirty-one (48%) post-COVID subjects reported experiencing gastrointestinal symptoms during SARS-CoV-2 infection (Table 1). Eight volunteers (11%) had confirmed exposure to SARS-CoV-2 by serological methods (when the samples were collected when vaccines were unavailable) but were asymptomatic. Factors affecting gut microbiota between the two groups, including preexisting comorbidities such as hypertension, hypothyroidism, irritable bowel disease, and chronic respiratory diseases, were similar between post-COVID individuals and controls (Figure 1b). Dietary habits were also analyzed (Table 2) and show similarity between the groups (Figure 1c). The use of antibiotics (three months before and during SARS-CoV-2 infection) was reported in 24 (33%) post-COVID subjects and 7 (12%) control subjects, with significant differences between both groups (Figure 1d). Notably, all fecal samples tested negative for SARS-CoV-2 nucleic acid by RT-qPCR at the time of collection (Figure 1e).

**Table 1.** Clinical and demographic characteristics of all human subjects in the study.

Parameters	Non-COVID-19	Asymptomatic	Symptomatic
**Gender, n (%)**	n = 59 (45.0%)	n = 8 (6.1%)	n = 64 (48.8%)
Male	19 (32.2%)	2 (25.0%)	24 (37.5%)
Female	40 (67.8%)	6 (75.0%)	40 (62.5%)
**Age Group, n (%)**			
<18 years	3 (5.1%)	-	2 (3.1%)
18–39 years	43 (72.9%)	3 (37.5%)	45 (7.3%)
40–49 years	5 (8.5%)	2 (25.0%)	11 (17.2%)
50–59 years	7 (11.9%)	3 (37.5%)	5 (7.8%)
>60 years	1 (1.7%)	-	1 (1.7%)
**Body Mass Index, n (%)**			
Underweight	2 (3.4%)	1 (12.5%)	4 (6.2%)
Normal weight	29 (49.15%)	3 (37.5%)	31 (48.4%)
Overweight	19 (32.2%)	3 (37.5%)	20 (31.2%)
Obesity class I	4 (6.8%)	-	8 (12.5%)
Obesity class II	3 (5.1%)	-	1 (1.6%)
**Symptoms during COVID-19, n (%)**			
Headache	-	-	47 (73.4%)
Fatigue	-	-	45 (7.3%)
Cough	-	-	40 (62.5%)
Anosmia	-	-	39 (6.9%)
Coryza or stuffy nose	-	-	37 (57.8%)
Myalgia	-	-	37 (57.8%)
Dysgeusia	-	-	36 (56.2%)
Fever ≥37,8°C	-	-	29 (45.3%)
Chills	-	-	21 (32.8%)
Sore throat	-	-	20 (31.2%)
Dyspnea	-	-	12 (18.7%)
Conjunctivitis	-	-	3 (4.7%)
**Gastrointestinal symptoms, n (%)**			
Diarrhea	-	-	22 (34.4%)
Appetite loss	-	-	19 (29.7%)
Nausea	-	-	11 (17.2%)
Abdominal pain	-	-	7 (1.9%)
Vomiting	-	-	3 (4.7%)

**Table 2.** Food characteristics used for the feeding score.

Group	Food group	Characteristics
A	**Ultra-processed food**	Chocolate, ice cream, pudding, mousse, cereal bar
		Sausage, salami, bologna, sausage, hamburger, turkey meat, chorizo
		Mayonnaise, margarine, whipped cream
		Ready sauces, frozen and ready-to-heat products
		Breads, cakes and cookies
		Pizza, French Fries, Chips, Instant Noodles
B	**Fiber source foods**	Fruits (Papaya, pear, grape, mango, guava, tangerine, pineapple, plum, watermelon, avocado, jabuticaba, acerola)
		Vegetables (Pumpkin, carrot, okra, chayote, cucumber, pepper, eggplant, pumpkin, cabbage, cauliflower, broccoli, radish, green beans, potato, Lettuce, cabbage, chicory, taioba, mustard, spinach, watercress, chard)
C	**Functional foods**	Beans, soy
		Oatmeal, honey, roll, curd
		Banana, apple, orange, lemon
		Tomatoes, beets, sweet potatoes, yacon potatoes, sauerkraut
		Soluble fiber (Garlic, Onion, Garlic)
		Probiotics (Yakult, Activia, Actimel, Kefir, Chamyto, Simfort)

Gut microbiota composition was analyzed by 16S sequencing in 44 fecal samples, prioritizing the sampling of paired subjects from the same household (15 families) see samples workflow in (Figure S1B). We attempted to sample paired subjects from the same family because human-associated microbiota communities vary across individuals, but cohabiting family members share a similar microbiota.^16,17^ Analysis of the gut microbiota revealed similar profiles between individual samples from controls and post-COVID subjects (Figure 1f). β-Diversity (weighted UniFrac distances) and α-diversity metrics (Shannon, Simpson, and Chao1 indices) showed no significant differences in the gut microbiota between the groups, indicating similar taxonomic diversity (Figure 1f).

To explore the possible impact of SARS-CoV-2 infection on the gut microbiota beyond its composition, we evaluated the frequency of cultivable *Enterobacteriaceae –* due to their importance as pathobionts that serve as a reservoir of antimicrobial resistance genes of clinical interest, comparing the total number of colony-forming units (CFU) in the feces between controls and post-COVID subjects. Despite similar CFU numbers between the two groups (Figure 1g), post-COVID subjects had a higher percentage of *Enterobacteriaceae* strains with drug-resistant (45% DR) and multidrug-resistant (23% MDR) phenotypes when compared to controls (39% DR and 13% MDR) (Figure 1h). We identified an increased prevalence of antimicrobial resistance (AMR) *Klebsiella* spp. among all Enterobacteriaceae strains assessed in post-COVID subjects compared to controls (Figure S1C). The *Klebsiella* strains were predominantly resistant to quinolones (64%), aminoglycosides (100%), and sulfonamides (91%) (data not shown), which were not associated with intrinsic resistance in this bacterial genus. Overall, these findings show increased AMR in the *Enterobacteriaceae* community of the gut microbiota post-COVID, which might be partly due to the increased overuse of antibiotics treatment in this subject group but could also be a direct effect of SARS-CoV-2 infection, although the mechanisms remain unclear.

### Post-COVID microbiota-induced alterations in the gut of microbiota humanized mice

**Figure 2.**
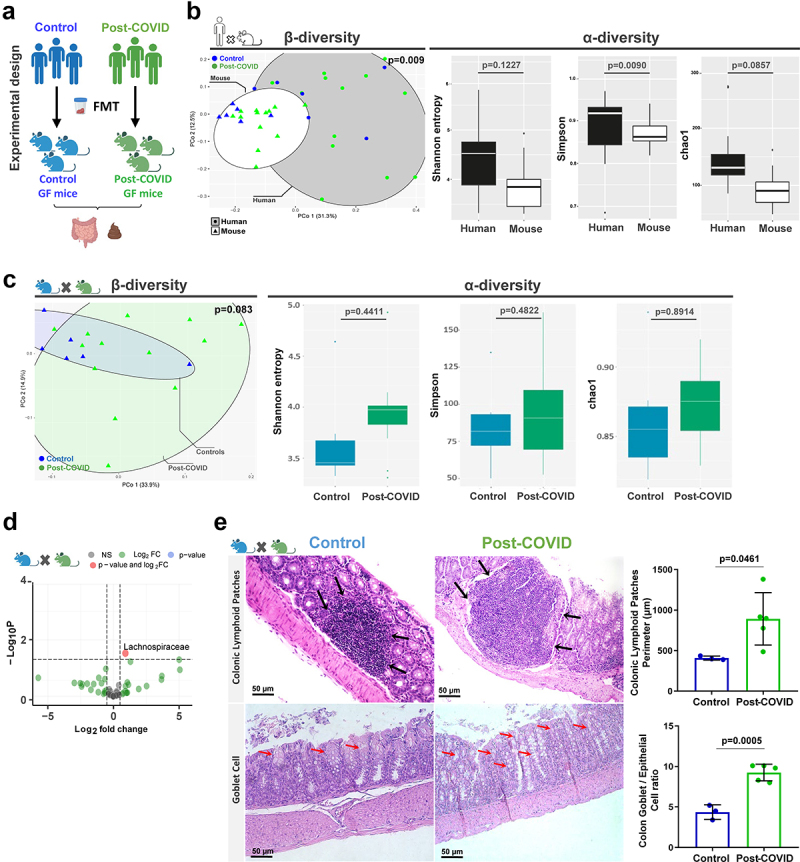
Control and post-COVID fecal microbiota transplant and effects on the gut of post-COVID mice. (a) experimental design: control (*N* = 8) and post-COVID (*N* = 14) mice received fresh feces from donors, and then analyzes of the gut microbiota and colon histology were performed 12 days after FMT. (b) 16S rRNA sequencing and comparison of the gut microbiota composition between human donor and mouse that received FMT. Principal Component analysis based on weighted Unifrac distances, a β-diversity index. α-diversity analysis based on Shannon, Simpson, and Chao1 indexes (donors *N* = 19; mice *N* = 19). (c) 16S rRNA sequencing of gut microbiota of control and post-COVID mice after FMT. β-diversity and α-diversity (*N* = 19). (d) Differential bacterial abundance in feces of control and post-COVID mice, *Lachonospiraceae* (*p* = .0300) (*N* = 19). (e) histological alterations in the large intestine in mice that received FMT. Black arrows indicate increases in Colonic lymphoid patches (*N* = 8). Red arrows Graphs showing the Colonic lymphoid patches Perimeter and the ratio between Goblet cells and epithelial cells in the colon. Statistical analysis: Wilcoxon test was used in B and C, PerMANOVA pairwise test was used in B and C, unpaired Student’s-*t* test was used in D, and Wald test was used in E. Data are shown as mean and standard deviation (SD). All results are representative of three independent experiments. See also Figure S2.

To understand the direct contribution of post-COVID microbiota to the host, we performed human fecal microbiota transplantation (FMT) in germ-free mice. Fecal samples were harvested from controls and post-COVID volunteers (Figure 2a), prioritizing samples belonging to the same family (co-housing human fecal samples) (Figure S1B) to minimize differences in the gut microbiota caused by other environmental factors. FMT was performed for each human microbiota donor transfer to individual germ-free mice (Figure S2A). After FMT, mice were housed for 10–12 days to stabilize the human-derived microbiota. This protocol was based on a previous study by our group, where we observed reversal hyporesponsiveness to inflammatory stimuli in germ-free mice 7 days after fecal transplantation.^18–20^ Nevertheless, there is no scientific consensus on the best methodology and timing of colonization for FMT in GF mice.

We evaluated human donor microbiota engraftment by first comparing the differences in β and α-diversity observed between fecal samples from human donors and recipient mice (Figure 2b). We analyzed engraftment levels by analyzing the percentage of bacterial groups from human donors retained in their respective recipient mice, we observed a level of engraftment between 45 and 75% and no difference between control and post-Covid groups (Figure S2B). During the engraftment, control and post-COVID mice presented similar mild diarrhea (commonly described during the first 5 days post-colonization) but without weight loss or any other observable alterations (data not shown). In addition to the differences between microbiota from human donors and recipient germ-free mice, transplanted humanized mice showed similarities between controls and post-COVID groups for both β- and α-diversity (Figure 2c). When comparing the composition and abundance of individual bacterial groups in the gut microbiota of transplanted humanized mice, the *Lachnospiraceae* family was significantly increased post-COVID compared to the control (Figure 2d). Although *Lachnospiraceae* were present in the inoculum of feces from donors, there were no differences between the control and post-COVID subjects (data not shown).

FMT can be used to demonstrate the direct effect of the gut microbiota on physiological processes. Therefore, we analyzed the impact of human microbiota in the gut of humanized microbiota (HM) mice compared to those that received feces from post-COVID *versus* control subject donors. HM mice that received feces from post-COVID subject donors did not show structural changes in the small intestine compared to controls but exhibited augmented cecal patches and an increase in goblet cells in the colon (Figure 2e).

To gain insights into the role of gut microbiota from post-COVID donors that may compromise intestinal homeostasis and influence systemic inflammation, we collected blood from human donors. We observed higher levels of the intestinal fatty acid-binding protein (I-FABP), a marker of epithelial damage (Figure S2C), suggesting disruption of gut epithelial barrier integrity in post-COVID subjects. However, this did not lead to increased translocation of gram-negative bacteria from the gut, because we did not observe differences in serum LPS levels between the groups (Figure S2D). Consistent with previous findings,^21^ the levels of commensal microbial metabolites (acetate, propionate, butyrate) were reduced in the fecal samples of post-COVID subjects compared to their paired controls (Figure S2E-G). These findings suggest that post-COVID subjects had signs of intestinal epithelial injury with increased circulating I-FABP levels that might influence extraintestinal organs.

### Post-COVID gut microbiota induces histological changes in the lung of humanized microbiota mice

**Figure 3.**
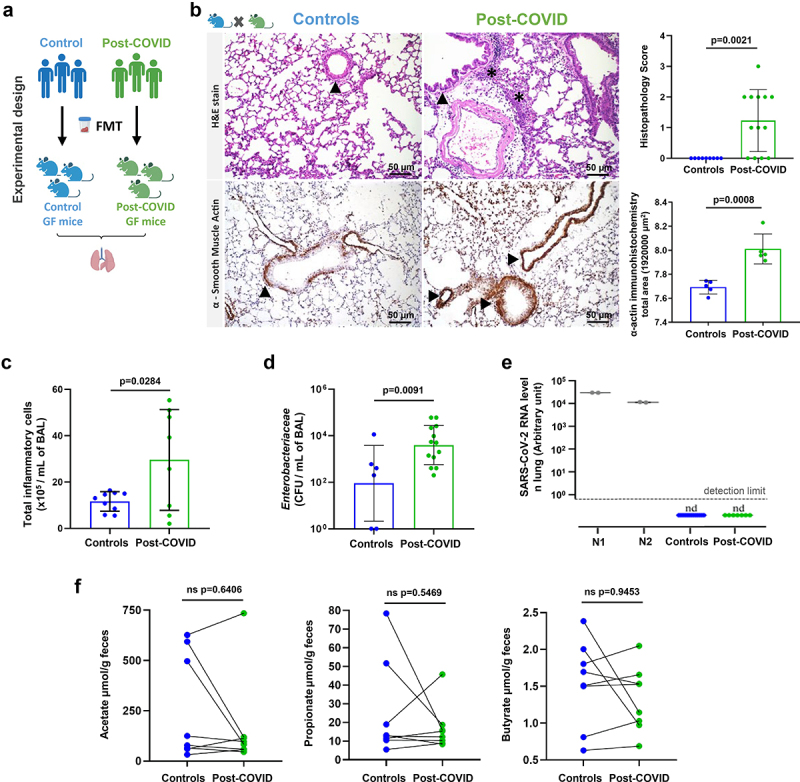
Post-COVID gut microbiota induce lung alterations in HM mice. (a) experimental design: germ-free mice received fresh feces from control (*N* = 8) or post-COVID (*N* = 14) donors and lung tissue and bronchoalveolar lavage were assessed 12 days after FMT. (b) H&E staining: the histopathological lung alterations induced by FMT to HM GF mice. Graph showing the histopathological score of airways, vascular and parenchymal inflammation in control and post-COVID mice lungs. Arrowheads indicate lung airways. Asterisks indicate inflammatory infiltrates. Scale bar: 50 µm. 20X objective (*N* = 22). α-SMA immune-staining: lung samples from HM GF mice and graph showing the morphometrical analysis of muscular layer changes. Ten images of the muscular layer of each animal were acquired with a 40X objective. Arrowheads indicate the immunostained area (*N* = 10). (c) total number of cells in bronchoalveolar lavage (*N* = 19). (d) cultivable *Enterobacteriaceae* load in bronchoalveolar lavage (*N* = 19). (e) RT-qPCR for SARS-CoV-2 in the lungs of control and post-COVID mice (*N* = 22). (f) paired analysis of feces acetate, propionate, and butyrate levels in control and post-COVID HM GF mice (*N* = 16). Statistical analysis: unpaired Student’s *t*-test was used in B, C and D. Wilcoxon matched-pairs signed rank test was used in F. Data are shown as mean and standard deviation (SD). All results are representative of three independent experiments.

To explore the systemic impact of post-COVID gut microbiota on extra-intestinal organs, we next assessed the effects of FMT on the lungs of recipient HM mice 12 days after transplantation (Figure 3a). We found foci of inflammatory infiltrates, mostly neutrophils, in both perivascular/peri-bronchial regions and increased histopathology scores in the lungs of mice receiving feces from post-COVID patients (Figure 3b). In addition, post-COVID mice showed increased expression of α-smooth muscle actin (α-SMA), indicative of physiological dysfunction in the lungs (Figure 3b). In addition, they had increased inflammatory cells in the bronchoalveolar fluid (BAL) compared to controls (Figure 3c). Cultivable *Enterobacteriaceae* in the BAL from post-COVID mice were found at higher levels than in control animals (Figure 3d), suggesting increased translocation of bacteria from the gut to the lung, which may account for the observed phenotype. Notably, SARS-CoV-2 nucleic acid was not detected in the lung tissues (Figure 3e), which corroborates the absence of the virus in the fecal samples used for the initial transplant (Figure 1e). We evaluated the levels of SCFAs in the feces of FMT mice and did not observe significant differences between controls and post-COVID mice (Figure 3f).

Overall, our data indicate that transfer of fecal samples from post-COVID subjects induced lung inflammation in recipient mice in the absence of SARS-CoV-2, suggesting that the intestinal microbiota modified by COVID-19 causes this phenotype.

### Fecal transplantation from post-COVID human volunteers to HM mice impairs host pulmonary defense

**Figure 4.**
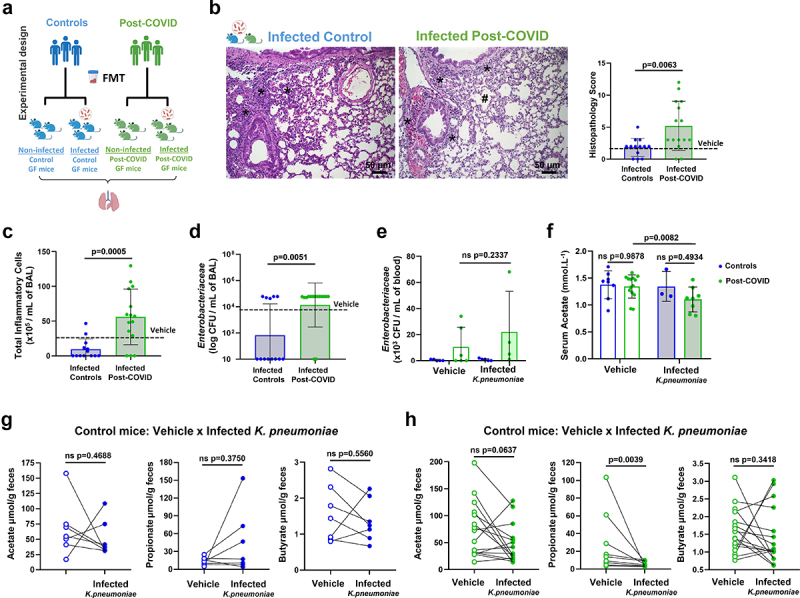
FMT from post-COVID patients impacts the gut-lung axis and increases susceptibility to *K. pneumoniae* B31 lung infection. (a) experimental design: germ-free mice received fresh feces from control or post-COVID donors and were infected with *K. pneumoniae* B31 (*K. pneumoniae*: control *N* = 13, post-COVID *N* = 15) or received saline (vehicle), and lung tissue, bronchoalveolar lavage and serum SCFAs levels were assessed. (b) histological alterations in the lung of post-COVID mice infected by *K. pneumoniae* B31 and a graph showing the histopathological score of the airway, vascular and parenchymal inflammation in control and post-COVID mice lungs (*N* = 28). Asterisks indicate inflammatory infiltrates. Hash marks areas of emphysema. Scale bar: 50 μm. 20X and 40X objective. (c) total number of inflammatory cells in bronchoalveolar lavage (BAL) (*N* = 28). (d) total numbers of *Enterobacteriaceae* in BAL (*N* = 28) and (e) blood (*N* = 28). (f) serum acetate levels (mmol.L^−1^) in vehicle and *K. pneumonia-*infected HM mice (*N* = 33). (g) paired analysis of fecal acetate, propionate, and butyrate levels between vehicle and infected mice that received feces from the same control donor (*N* = 14). (h) paired analysis of fecal acetate, propionate, and butyrate levels between vehicle and infected mice that received feces from the same post-COVID donor (*N* = 30). Statistical analysis: unpaired Student’s *t*-test was used in B and C, Mann-Whitney test was used in D. Two-way ANOVA with Tukey’s tests was used in E and F. Wilcoxon matched pairs signed rank test was used in G and H. Data are shown as mean and standard deviation (SD). All results are representative of three independent experiments.

We hypothesized that the consequences of post-COVID fecal transplant-induced lung alterations could further impact host defense and favor secondary infections. To validate this hypothesis, we intranasally infected HM mice with a multidrug-resistant strain of *Klebsiella pneumonia (Kp)* (Figure 4a). We compared *Kp* lung infection in control *versus* post-COVID mice and observed higher pathological changes in the perivascular, peri-bronchial, and parenchymal regions characterized by emphysema-like areas in the lungs of infected post-COVID mice (Figure 4b). These changes were associated with intense inflammatory cell infiltration in the BAL of these mice compared to the infected control mice (Figure 4c). Despite this, the recruitment of inflammatory cells to the lungs was inefficient for the clearance of the bacteria because we harvested similar CFU levels of *Kp* in the lungs of both control and post-COVID-infected mice (Figure 4d). We also observed an increase in *Enterobacteriaceae* CFU numbers in the blood of both post-COVID *Kp-*infected and non-infected (vehicle) mice suggesting systemically translocation of gram-negative bacteria in post-COVID mice (Figure 4e). Our group and others have demonstrated that acetate, a gut microbiota metabolite, contributes to the control of pulmonary infection induced by the pathogen *Klebsiella pneumonia* in mice.^22–24^ Accordingly, we observed reduced serum acetate levels in post-COVID *Kp-*infected mice compared to those in post-COVID non-infected (vehicle) mice (Figure 4f). When we correlated the fecal levels of SCFAs in paired mice, we did not observe significant differences in non-infected (vehicle) control mice and their paired *Kp*-infected mice (Figure 4g). However, we noted a propionate reduction in the fecal samples of post-COVID mice *Kp*-infected compared to their non-infected (vehicle) post-COVID donor-paired mice (Figure 4h).

Overall, our data indicate that transplantation of post-COVID feces affected the lungs of recipient mice in the absence of SARS-CoV-2 and contributed to the impairment of the host lung defense against bacterial infection.

### Post-COVID gut microbiota induces memory impairment and hippocampus changes and can be partially reversed with probiotics

**Figure 5.**
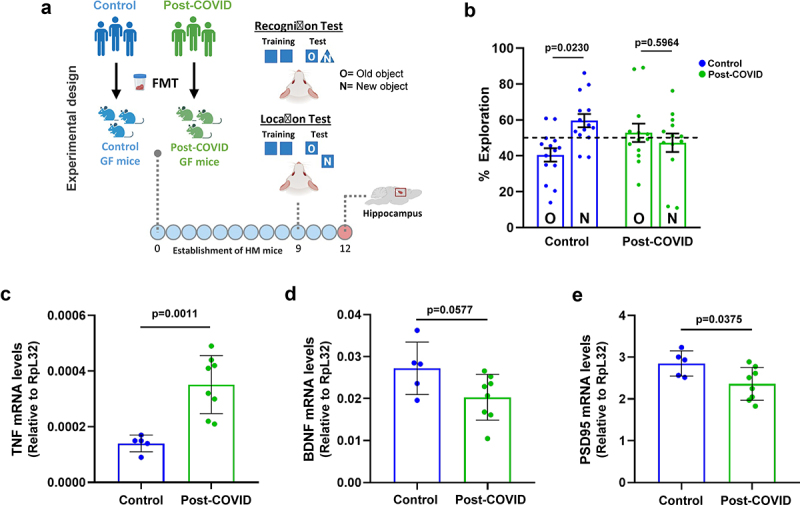
FMT from post-COVID patients induces cognitive alterations in HM mice. (a) experimental design: germ-free mice received fresh feces from control (*N* = 14) or post-COVID (*N* = 15) donors, and underwent cognition (object location and recognition) tests nine days later. Following behavioral analysis, their hippocampus was subjected to mRNA expression. (b) percentage of exploration time for the new object (N) or the one remaining unmoved (O) in the location test relative to a total exploration time (*N* = 29) 9 days after FMT. Quantification of the expression, by RT-qPCR, of (c) TNF (*N* = 13), (d) BDNF (*N* = 13), and (e) PSD95 (*N* = 13) in the hippocampus 12 days after FMT. Statistical analysis: One sample *t* test against the hypothetical value of 50% and unpaired Student’s *t* test was used in B. Data are shown as mean and standard deviation (SD). All results are representative of two independent experiments.

In addition to lung alterations, accumulating reports have found that post-COVID sequelae are commonly associated with brain dysfunction.^12–25–27^ To further explore the causal effects of gut microbiota on brain alterations, HM germ-free mice that received FMT from post-COVID and control subjects were subjected to cognitive behavioral tests (Figure 5a). Post-COVID mice showed memory impairment in the recognition and location tests compared to control mice (Figure 5b). Control mice showed significantly more interaction with a novel object in the cage than post-COVID mice. Based on these phenotypes, we analyzed inflammatory markers in the hippocampus of post-COVID mice, which could indicate a possible connection with neuroinflammation. We observed increased mRNA expression of the pro-inflammatory cytokine *tnf* but lower levels of neuroprotective factors such as *bdnf* and *psd-95* in post-COVID mice than in controls (Figure 5c–e). Together, these results suggest that alterations in gut microbiota and inflammation caused by COVID-19 can directly cause changes in brain cognition.

**Figure 6.**
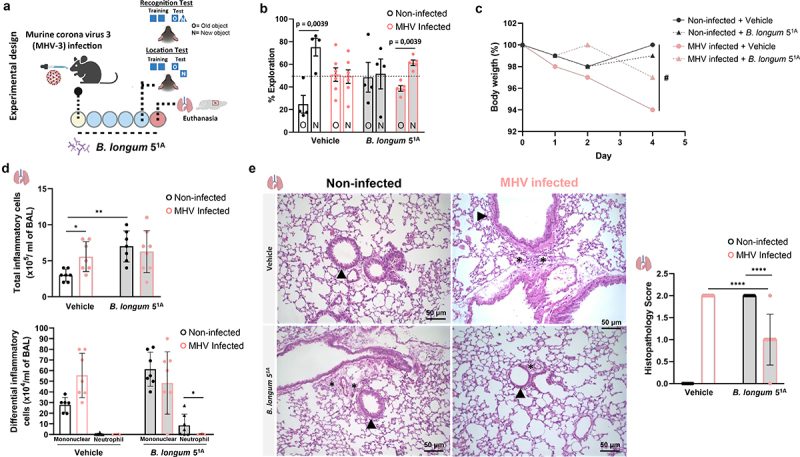
The mouse model of MHV-3 infection showed memory impairment in object recognition and location tests, and treatment with *B. longum* 5^1A^ reversed the cognitive alterations. (a) experimental design: C57BL/6 non-infected and MHV-3 infected and treated with probiotic *B. longum* 5^1A^ (vehicle: non-infected *N* = 4; MHV-3 infected *N* = 7; *B. longum* 5^1A^: non-infected *N* = 4; MHV-3 infected *N* = 5), and subjected to behavioral (object location and recognition) tests 4 days later. (b) percentage of exploration time for the new object (N) or the one remaining unmoved (O) in the location test relative to a total exploration time (*N* = 20). (c) Body mass over time was measured daily throughout the experiment. (# significant main effect of MHV infection) (*N* = 29). (d) total number of inflammatory cells in bronchoalveolar lavage (BAL) (*N* = 29). Differential inflammatory cells number in bronchoalveolar lavage (BAL) (*N* = 29). (e) H&E staining: histological alterations in the lung of MHV-3 infected mice and treated with probiotic B. longum 51A. Graph showing the histopathological score of the airway, vascular and parenchymal inflammation in control and post-COVID mice lungs (*N* = 29). Arrowheads indicate lung airways. Asterisks indicate inflammatory infiltrates. Scale bar: 50 μm. 20X objective. Statistical analysis: One sample t-test against the hypothetical value of 50% was used in B. Three-way repeated measures ANOVA with Tukey’s test was used in C. Two-way ANOVA with Student-Newman-Keuls test was used in D and E. Data are shown as mean and standard deviation (SD).

To further investigate and test the potential of microbiota-based interventions as targets to prevent memory impairment induced by direct coronavirus infection, we used a β-coronavirus murine model of lung infection.^28^ This experimental murine model of mouse hepatitis virus (MHV-3) mimics human COVID-19 inflammatory manifestations in mice, and we also observed that infected animals had memory impairment using the object location test. Using this model, we tested the potential of microbiome-based interventions by administering the probiotic *Bifidobacterium longum* 5^1A^ (Figure 6a). The particularity of choosing the probiotic *B. longum* 5^1A^ strain is the ability to produce high levels of SCFAs, which has beneficial effects in controlling host inflammation, particularly in the lung as previously demonstrated by our group.^23,29^ Treatment with *B. longum* 5^1A^ prevented memory impairment induced by MHV-3 infection in mice (Figure 6b). The probiotic treatment also reduced the weight loss caused by the murine coronavirus infection (Figure 6c). There is no alteration in the total numbers and/or differentiation of any inflammatory cells in both probiotic-treated bronchoalveolar fluid (BAL) in either probiotic-treated or not in MHV-3 infected mice (Figure 6d). Notably, treatment with *B. longum* 5^1A^ to non-infected mice *per si* increased inflammatory infiltration of cells in the BAL (Figure 6d). Still, treatment with *B. longum* 5^1A^ reduced the lung tissue inflammation characterized by a reduction in the foci of inflammation in perivascular and peribronchial regions induced by MHV-3 infection, as shown in the histopathology, and measured by inflammatory score (Figure 6e). To test the effect of the SCFA, acetate, on MHV-3 condition, we treated mice orally with acetate (Figure S6). However, we did not find any decrease in the symptoms while observing minor protection in the survival MHV-3-infected mice treated with acetate (Figure S6A).

Statistical analysis: One sample *t* test against the hypothetical value of 50%. Data are shown as mean and standard deviation (SD).

Altogether, our data indicate that alterations in the gut microbiota contribute to the post-COVID disruption of hippocampal function, leading to cognitive impairment, which might be prevented or attenuated via microbiome-based interventions.

## Discussion

Among pathophysiological responses triggered by SARS-CoV-2 infection, several studies showed associations between gastrointestinal symptoms and altered gut microbiota in COVID-19 during and after the infection.^12–30^−^33^ However, our study is the first to show a causal effect of gut microbiota alterations on post-COVID sequelae. Our findings confirm previous data that SARS-CoV-2 infection is associated with the spread of antimicrobial resistance in the gut microbiota.^34^^35^^36^^37^^–^34^39^ In fact, we observed an increase in AMR *Enterobacteriaceae* in the gut microbiota of post-COVID individuals who were either asymptomatic or had mild symptoms of COVID-19. This is especially surprising, as COVID-19-related AMR spread has been mainly associated with moderate/severe cases and hospitalized individuals.^38^ Although the widespread use of antimicrobials during COVID-19 and overuse between our post-COVID and control volunteers may explain the increased AMR spread,^40–43^ we cannot exclude the direct impact of SARS-CoV-2 infection on increasing AMR through the microbiome. Notably, we found a predominant increase in AMR *Klebsiella* sp. in post-COVID gut microbiota subjects. This bacterium belongs to the ESKAPE group (*Enterococcus faecium, Staphylococcus aureus, Klebsiella pneumoniae, Acinetobacter baumannii, Pseudomonas aeruginosa, and Enterobacter species*) of AMR pathogenic bacteria that are responsible for most nosocomial pneumonia in hospitalized patients with COVID-19.^44,45^ Moreover, post-COVID mice had worse lung injury, which compromised the lung immune response to *K. pneumoniae* B31 infection. Therefore, post-COVID microbiota transfer may have contributed to the impairment of the pulmonary immune system, inducing a greater susceptibility to infections caused by pathogens/pathobionts.^46–49^ Lung microbiota disruption is another relevant factor associated with pulmonary alterations observed in post-COVID mice is lung microbiota disruption.^50^ The lung microbiota is known to be susceptible to gut microbiota alterations, highlighting the relevance of the gut-lung axis.^50,51^ Although we did not evaluate the lung microbiota as a whole, we found increased cultivable *Enterobacteriaceae* in the lungs and pulmonary tissue damage in mice with post-COVID gut microbiota, but not in the controls. These findings raise questions about the components in the fecal samples that induce such alterations after transplantation and which mechanisms are involved in the gut-lung connection.

The next step in our investigation was to deepen our understanding of the effects triggered by post-COVID microbiota by exploring other host tissues. Therefore, the connection between the gut and brain is well established,^52^ and most recently, it has been described as the lung-brain axis.^53,54^ Our finding that post-COVID microbiota can induce memory impairment in transplanted mice suggests a possible connection with the neurological outcomes of post-acute COVID-19 in humans.^55,56^ Although our study did not assess the role of the lung microbiota in neurological disorders, clinical studies have shown differences in the lung microbiota between cognitively impaired individuals *versus* controls.^57^ Previous studies suggested that even minimal alterations in the lung microbiome can affect the central nervous system, although significant changes in gut microbiota were necessary.^58^ Thus, lung alterations induced by the microbiota in post-COVID human donors in post-COVID mice may be associated with neurological outcomes.

Gut microbiome production of metabolites, particularly short-chain fatty acids (SCFA), is an effective mechanism that supports the gut-lung and gut-brain axis.^14,59^ SCFAs are also known to play a role in the development of the immune system and lung mucosal function, thereby protecting against infections and pulmonary damage.^59^ Furthermore, SCFA can also modulate blood-brain barrier integrity and inflammatory responses in microglia,^58^ and clinical and experimental studies have associated reduced SCFA levels with Alzheimer’s disease.^60^ We observed reduced acetate, propionate, and butyrate levels in the feces of post-COVID donor subjects and decreased acetate levels in post-COVID *Kp*-infected mice. Thus, this decrease in SCFA levels could affect the gut-lung and gut-brain connections and help explain neurological sequelae and the increased susceptibility to pulmonary coinfections observed post-COVID.

We also observed apparent differences in the systemic levels of host factors, such as I-FABP, indicating a loss of intestinal homeostasis in post-COVID subjects compared with controls. Indeed, I-FABP is a relevant prognostic biomarker that is positively correlated with a worse prognosis in COVID-19.^61^ Thus, although no significant differences in α- and β-diversity were observed in the gut microbiota of post-COVID and control subjects, functional changes were observed in both patients and mice. Notably, enrichment of the *Lachnospiraceae* family in post-COVID mice is corroborated by previous findings, where subjects with COVID-19 had a higher prevalence of this group of bacteria.^62,63^

We observed increased TNF expression in the hippocampus of mice with post-COVID gut microbiota, suggesting neuroinflammatory responses in the central nervous system. A similar effect was observed in a colitis model, where systemic-driven hippocampal TNF expression was associated with memory impairment, which was abolished upon restoration of the gut microbiota^64^. Furthermore, we found reduced expression of the neuroplasticity markers BDNF and PSD-95 in the hippocampi of post-COVID mice. This impairment in neuroplasticity was previously observed when feces from transgenic Alzheimer’s disease mice transferred their cognitive phenotype to recipient mice.^65^

As a proof-of-concept for the potential use of microbiome-based approaches for post-COVID sequelae, we used a probiotic strain of *B. longum* 5^1A^ to assess a mouse model of coronavirus infection. Here, we used the probiotic *B. longum* 5^1A^ isolated from the gut microbiota of healthy children, which can produce high levels of SCFAs and has beneficial effects in controlling inflammation at a systemic level, especially in the lung.^23,29,66^ We observed neuroprotective effects of *B. longum* 5^1A^ which prevented memory impairment after lung infection with a murine coronavirus. Although the effects of *B. longum* 5^1A^ should be explored further, these data suggest that therapies targeting the gut microbiota are promising approaches for treating post-acute COVID-19 consequences.^67^

Our study indicates a direct connection between altered gut microbiota and post-COVID symptoms.^15^ However, some limitations of this study should be considered. First, our study had a relatively small sample size and compared humans that were not perfectly age- and sex-matched. Other factors, such as gut viromes, were not assessed in our study but were associated with clinical outcomes of COVID-19.^8^^,^^68–70^ Finally, SARS-CoV-2 antigens can persist for long periods in the intestine, feces, and gut tissues, boosting immune responses that may fuel post-COVID symptoms.^71^ However, we did not detect SARS-CoV-2 in the feces of donors or lungs of humanized microbiota mice.

Collectively, our results suggest a direct connection between the long-term effects of COVID-19 and alterations in the gut microbiota after the clearance of SARS-CoV-2 infection. Our findings emphasize the need to define how the gut microbiota is affected by SARS-CoV-2 infection, even in individuals who do not have severe symptoms. This is especially important, given the increased AMR in the gut microbiota of patients previously infected with SARS-CoV-2. AMR has spread at alarming rates, and the effects of the COVID-19 pandemic on the gut microbiota resistome could have a major additional contribution.

## Materials and methods

### Study design

This cross-sectional study consisted of two steps:**1**. Fresh feces and blood were collected from post-COVID and health volunteers (control), and a survey consisting of clinical symptoms during COVID-19, medication use, and lifestyle questions was administered (Figure S1A); **2**. We developed humanized microbiota (HM) mice by performing individual FMT from these donors into GF-recipient mice (Figure S2F). For the FMT, samples of subjects (Control and Post-COVID) from the same household (*n* = 6 families; in total *n* = 12 members) were prioritized, where one was post-COVID and the other was not infected (Control). Additionally, random samples of post-COVID individuals (*n* = 8) were added to the experimental group, totaling 19 samples. In addition to the second stage of the study, an experimental pulmonary infection induced by the multidrug-resistant *Klebsiella pneumoniae* B31 strain was performed (Figure 4a).

### Study subjects and sample collection

Seventy-two post-COVID volunteers (after nucleic acid amplification test [NAAT] or an antigen test confirmed SARS-CoV-2 infection) and fifty-nine control volunteers (SARS-CoV-2 NAAT or antigen and IgG/IgM negative) were included (Figure S1A). In the post-COVID group, sixty-four volunteers were symptomatic (collections were performed 1 to 4 months after infection), and eight were asymptomatic (classified after antigen test). All volunteers, controls, and post-COVID were between 15 and 60 years of age. The clinical spectrum of disease severity (mild and moderate) in post-COVID and the volunteers was classified according to the NIH COVID-19 Treatment Guidelines (National Institutes of Health, 2022). Vaccination for SARS-CoV-2 and positive serological test results at the time of sample collection were excluded. Samples were collected between October 2020 and April 2021 from Belo Horizonte, Minas Gerais, Brazil. Fecal samples were collected in a sterile container and immediately stored in a home refrigerator (4°C °C) for 12 h prior to analysis and FMT in the laboratory. All subjects consented to participate in this study with the approval of the Ethics Committee on Human Research of Universidade Federal de Minas Gerais (COEP) protocol 4.615.698.

### SARS-CoV-2 load in fecal samples

RNA extraction from fecal, human, and mouse samples was adapted from a previously published protocol.^72^ Briefly, fecal samples were diluted 1:5 (w:v) in guanidine, homogenized, and clarified by centrifugation (4,000x*g*, 20 min, 4°C). Viral RNA was purified using the QIAamp Viral RNA Mini Kit, following the manufacturer’s instructions. RT-qPCR for SARS-CoV-2 was performed using the one-step RT-qPCR Master Mix according to the CDC USA protocol (CDC, 2020), and primers for N1 and N2 (cat. no. 10006770) on a QuantStudio™ 7 Flex Real-Time PCR System platform (Applied Biosystems, USA). For analysis, amplification values of N1 or N2 viral targets with threshold cycle (Ct) below 40.0 were considered positive for SARS-CoV-2, and above 40 or indeterminate were considered undetectable, and the relative concentrations were expressed in arbitrary units. Fecal samples spiked with inactivated SARS-CoV-2 (stock titer 6.7 × 10^6^ PFU/mL) were used as a positive control at different dilutions.

### Gut microbiota composition analysis

DNA extraction of fecal samples – human and mouse – stored at -70°C was performed using QIAamp DNA Stool Mini Kits (Qiagen, USA) according to the manufacturer’s instructions. DNA was used as a template in the PCR amplicon, targeting the V3 and V4 hypervariable regions of the bacterial 16S rRNA gene. The Illumina 16S Metagenomic Sequencing Library Preparation protocol was used to prepare a 16S rRNA gene library. The 16S library was quantified using the Qubit dsDNA HS Assay Kit (Invitrogen, USA) and checked using a 2100 Bioanalyzer Instrument (Agilent Technologies, USA). The sample pool (4 nM) library was diluted to a final concentration of 8 pM and added to 20% (v/v) of 8 pM PhiX DNA (Illumina, USA), following the Illumina guidelines. Sequencing was performed using the Miseq reagent kit v3 (600 cycles) on the Illumina MiSeq platform and 2 × 300 bp (MSC v2.4), according to the manufacturer’s instructions (Illumina, USA). 16S rRNA gene sequence data were processed using the QIIME2 pipeline.^73^ First, sequenced reads were denoised with DADA2 and then processed by VSEARCH^74^ to filter chimeras and perform de novo clustering of valid sequences into OTUs with 97% sequence similarity. Next, MAFFT Fasttree was used to conduct phylogenetic analysis based on OTUs. α- and β-diversity were analyzed using the core-metrics-phylogenetic method built into QIIME2, and the Shannon and Simpson diversity indices and Chao1 were calculated; for β-diversity, Bray-Curtis and weighted UniFrac distances were calculated. OTUs were taxonomically classified using Naive Bayes classifiers trained with Silva v. 138, with 97% sequence similarity to full-length OTUs. Differential abundance was calculated using the ANCOM.^75^ A two-sided alternative Wilcoxon test was performed for alpha diversity parameters, while pairwise Permanova was performed for beta diversity parameters. The differential bacterial abundance was calculated using DESeq2^76^ and plotted as a volcano plot using the EnhancedVolcano package.^77^ The bacterial families with log2 Fold-change >0,5 and P-value ≤0.05, were considered statistically different between conditions. Statistical analyses were performed using the R statistical software environment (The R Foundation, Austria). The 16S rRNA gene amplicon sequencing library produced in this study was deposited in the NCBI SRA database under the project number PRJNA843134.

### Enterobacteriaceae *identification and antimicrobial resistance test*

Fresh fecal samples were homogenized (100 mg for each 1 mL of sterile 0.9% saline) and serially diluted (1:10). Subsequently, different dilutions were plated on MacConkey agar (Sigma, Germany) and incubated for 24 h at 37°C under aerobic conditions. The colonies were counted, and the data were expressed as the log_10_ of colony-forming units (CFU) per milligram of feces. *Enterobacteriaceae* colonies with different morphologies were isolated from the MacConkey agar. Pure colonies were suspended in sterile 0.9% saline at a 1.5 × 10^8^ CFU/mL concentration according to the 0.5 McFarland standard. Then, a sterile swab was soaked in the bacterial solution and inoculated by spreading on Mueller-Hinton agar plates (140 × 15 mm) (Merck, USA). After 15 minutes, a dispenser (Thermo Scientific, Remel™, USA) with 12 discs (Thermo Scientific, Oxoid™, USA) referring to β-lactam (amoxicillin-clavulanic acid, cephalosporin, ertapenem, meropenem, imipenem), aminoglycosides (amikacin, streptomycin, gentamicin), quinolones (ciprofloxacin, levofloxacin, norfloxacin), sulfonamide and folate inhibitors (sulfamethoxazole-trimethoprim) and macrolides (azithromycin) antibiotics were added to the inoculated plates. The plates were incubated at 37°C for 24 h. The presence or absence of bacterial growth inhibition zones was observed and measured to determine the resistance profile in: sensitive, intermediate, or resistant, according to the CLSI M100Ed31 guidelines. The resistance phenotype was determined according to the number of antimicrobial classes in which each strain presented resistance: resistant (1–2 antimicrobials) or multidrug-resistant (≥3 antimicrobials). Identification of *Enterobacteriaceae* strains was performed by Matrix Associated Laser Desorption-Ionization – Time of Flight (MALDI-TOF), using the FlexControl MicroFlex LT mass spectrometer (Brunker Daltonics, USA) as described before.^78^ Before identification, calibration was performed using the *Escherichia coli* DH5α test standard (Brunker Daltonics, USA).

### Laboratory animals

Male and female germ-free Swiss/NIH mice derived from a GF nucleus (Taconic Farms, USA), with ~ 8-weeks-old were used. They were maintained in flexible plastic isolators (Standard Safety Equipment Co., USA) using classical gnotobiology techniques at the Gnotobiology Laboratory of the Federal University of Minas Gerais (UFMG), Minas Gerais, Brazil, under controlled conditions (26°C, 12 h light/dark cycle) with steam sterilized food (Nuvilab, Brazil) and sterile water *ad libitum*. For the FMT experiments, germ-free mice were kept individually in sterile microisolator cages (UNO Roestvaststaal B.V., Netherlands) throughout the experiment to avoid cross-contamination and ensure the individual feces donor phenotype for the respective HM mice. Also, Male and female C57BL/6J mice, aged ~8 weeks old, obtained from the UFMG animal facility, were kept in plastic cages (Alesco, Monte Mor, Brazil) in a room with controlled conditions (26°C, 12 h light/dark cycle) with steam sterilized food (Nuvilab, Brazil) and sterile water *ad libitum*. All mouse procedures were performed in accordance with the guidelines of the Guide for the Care and Use of Laboratory Animals of the Brazilian National Council of Animal Experimentation (http://www.cobea.org.br/) and the Brazilian Federal Law 11.794 (October 8, 2008). The animal study was reviewed and approved by The Institutional Committee for Animal Ethics of the Federal University of Minas Gerais (protocol no. CEUA/UFMG 281/2020 and 55/2021).

### Human fecal microbiota transplant to GF mice

Fresh fecal samples were used for FMT, and samples from members of the same household were prioritized (*n* = 6 families; *n* = 12 members). In addition, randomized samples of post-COVID subjects were included (*n* = 8), totaling 19 samples (Figure S2F). Each sample was weighed and resuspended in 0.9% sterile saline (NaCl) solution (100 mg/mL). FMT was performed individually, and each animal received a sample from an individual donor volunteer. The fecal transfer was not sex-matched, but the proportion of male and female animals was the same in groups receiving fecal transfer from control or post-COVID subjects. A 100 μL aliquot was used for oral gavage of GF mice, with the same concentration and volume of feces and bacteria in all animals. After nine days, the experiments were performed to ensure a stable human microbiota population of GF mice when we were able to transfer human bacteria to GF mice, according to our group.^18,79^

### Histopathology and immunohistochemistry

Intestine and lung tissues from HM mice were collected and processed. For the intestinal morphometric analysis, images (20X objective) were acquired from hematoxylin and eosin (H&E) staining for quantification of the colonic lymphoid patches and globet cells, and the absolute number of colon globet cells per epithelial ratio and perimeter of lymphoid patches in the controls compared to post-COVID mice. For lung analysis, the inflammatory score was determined using hematoxylin and eosin (H&E)-stained slides, and airway, vascular, and parenchymal inflammation were evaluated, as previously described.^80,81^ For immunohistochemical analysis, lung tissue slides from control and post-COVID HM mice were immunostained. Briefly, the slides were incubated with primary anti-α-actin antibody (1:500) (DAKO, USA) overnight at 4°C. The primary antibodies were detected using an anti-mouse/anti-rabbit detection system (Novolink Polymer Detection System; Leica Biosystems, UK) according to the manufacturer’s instructions. The sections were counterstained with diluted Harris Hematoxylin solution and permanently mounted using Entellan (Merck, USA). For the morphometric analysis, images (20X objective) were acquired from immunolabeled α-actin to quantify the muscle layer of the lung section. For intestine and lung morphometric analyses, we used the ImageJ 1.52 program (NIH, USA). All analyses were performed under a light microscope by a pathologist blinded to the experiment.

### Bronchoalveolar lavage collection and analysis

After anesthetizing and euthanizing the mice (ketamine/xylazine, 180 and 12 mg/kg, respectively), bronchoalveolar lavage (BAL) was performed by inserting and collecting 1 mL of sterile phosphate-buffered saline (PBS) through a 20-gauge catheter in a 1-mL syringe. The *Enterobacteriaceae* quantification was quantified by plating an aliquot (100 µL) in MacConkey medium and incubating under aerobic conditions for 24 h at 37°C. Colonies were counted and expressed as CFU per mL of BAL. For airway inflammatory cell counts, the remaining BAL was centrifuged and resuspended in 100 µL of saline, and total leukocytes were quantified by Neubauer chamber counting.

### Klebsiella pneumoniae *infection*

*K. pneumoniae* B31, a clinical isolate with an AMR profile,^82^ was kindly provided by Prof. Vasco Azevedo, Laboratory of Cellular and Molecular Genetics at ICB/UFMG. Intratracheal infection was induced as previously described.^23^ Briefly, anesthetized animals were exposed to the trachea and 25 μL of the suspension containing 1 × 10^6^ CFU/mL of *K. pneumoniae* B31, or sterile saline for vehicle control animals, was administered with a 26-gauge needle.

### Short-chain fatty acid (SCFA) assays

Human and mouse fecal samples were suspended in 1% phosphoric acid (1:6 weight:volume) (Merck, USA), vortexed, and centrifuged (20,000x*g*, 30 min, 4°C). Supernatants were filtered (0.22 µm) and injected directly into an HPLC system with an ionic exchange resin column 300 × 7.8 mm (Sigma, Germany) at 30°C with a Micro-Guard cation H^+^ cartridge (Sigma, Germany) and detector set at 210 nm. The flow rate was 0.5 mL/min for 35 min, changed to 0.7 mL/min until the end of the 55 min chromatographic run. Serum samples were diluted in formic acid (1 mol.L^−1^), and internal standard, 2-ethyl-butyric acid 1 mol.L^−1^, (Sigma, Germany) was added, in proportions 5:5:1 respectively, followed by vortexing and centrifugation (12,000x*g*, 30 min, 4°C). Next, supernatants were injected into a Gas Chromatograph – FID (Agilent, USA), with an HP-FFAP column 19091F–105 (Agilent, USA), 50 m × 0.20 mm × 0.33 µm, and the detector set at 240°C. chromatographic conditions were 60°C for 0.5 minutes, heating at 8°C.min^−1^ to 180°C for 1 min, with a new heating rate of 20°C.min^−1^ at 240°C for 7 min. The total run time was 26.5 minutes. Seven-point external calibration curves were adopted to quantify fecal and serum samples using analytical-grade SCFA (Sigma, Germany) as standards.

### Mouse behavioral tests

The tests were performed in a 30 (w) × 30 (d) × 45 (h) cm arena, where each animal was allowed to freely explore for 5 min. The next day, the mice were subjected to a 5 min training session, during which they were placed at the center of the arena in the presence of two identical objects. The time spent exploring each object was then recorded. The test session was performed after 30 min by replacing one of the two identical objects with a new one in the object recognition paradigm or moving the training object to a new location in the new object location paradigm.^83^ The results were expressed as a percentage of time exploring each object or location, old (O) or new (N), concerning the total exploration time during the test session.

### Mouse hippocampus analysis

Total RNA from the hippocampus of post-COVID HM mice and controls was extracted using TRIzol reagent (Thermo Scientific, USA). cDNAs was synthesized and subjected to qPCR using Power SYBR Green Master Mix kits following the manufacturer’s instructions on the QuantStudio™ 7 Flex real-time PCR system platform (Applied Biosystems, USA). Primer sequences used are listed in Table 3. Gene expression changes were determined by the 2^–ΔCt^ method using ribosomal protein L32 for normalization.

**Table 3.** Primer sequences for RT-qPCR.

Gene Name	Forward primer (5’-3’)	Reverse primer (5’-3’)
*IL-1β*	TTGACGGACCCCAAAAGAT	GAAGCTGGATGCTCTCATCTG
*IL6*	GATGGATGCTACCAAACTGGA	CCAGGTAGCTATGGTACTCCAGAA
*TNF*^1^	TGCTGGGAAGCCTAAAAGG	CGAATTTTGAGAAGATGATCCTG
*TNF^2^*	GCTGAGCTCAAACCCTGGTA	CGGACTCCGCAAAGTCTAAG
*IFNβ*	GCCCTGTAGGTGAGGTTGATCT	AGCTCCAAGAAAGGACGAACAT
*PSD-95–Dgl4*	TCTGTGCGAGAGGTAGCAGA	AAGCACTCCGTGAACTCCTG
*BDNF*	ATGAAAGAAGTAAACGTCCAC	CCAGCAGAAAGAGTAGAGGAG
*RpL32*	GCTGCCATCTGTTTTACGG	TGACTGGTGCCTGATGAACT
MHV-3	CAGATCCTTGATGATGGCGTAGT	AGAGTGTCCTATCCCGACTTTCTC

^1^Primer used for RT-PCR in lung and gut samples; ^2^Primer used for RT-PCR in brain samples; IL, interleukin; TNF, Tumor necrosis factor; IFN, interferon; PSD-95–Dgl4, Post-synaptic density protein; BDNF, Brain-derived neurotrophic factor; RpL, Ribosomal protein; MHV-3, Murine Hepatitis Virus-3 nucleocapsid protein (N) gene.

### Mouse hepatitis virus-3 (MHV-3) infection

The β-coronavirus mouse hepatitis virus MHV-3 (3 × 10^1^ PFU), propagated in L929 cells or sterile saline for sham controls, was used to intranasally infect C57BL/6J mice as previously described.^28^ Some groups were treated with B. longum 5^1A^. The probiotic bacterium *B. longum* 5^1A^, from the culture collection of the Laboratory of Biotherapeutic Agents at ICB/UFMG was isolated from fecal samples of healthy children and cultivated as previously described.^23,84^ Briefly, mice were treated by oral gavage of a single 100 μL dose of suspension containing 1 × 10^9^ CFU/mL *B. longum* 5^1A^ or sterile saline every 48 h during the infection period.^23^ Four days after infection, behavioral tests were performed.

### Intestinal fatty acid-binding protein (I-FABP) assays

I-FABP was quantified by enzyme-linked immunosorbent assay (ELISA) in accordance with the manufacturer’s instructions (R&D Systems, USA) and as previously described.^61^

### Statistical analysis

Statistical analyses were performed using GraphPad Prism7 (GraphPad Software, USA) and R software v.4.2.2 (R Core Team, 2013). Graphs were produced GraphPad Prism7, Microsoft PowerBi (Microsoft Corporation, USA), or R software v.4.2.2. Data normality and homoscedasticity were tested using the Shapiro-Wilk and Levene tests, respectively. Data with a normal distribution were evaluated by Student’s *t* test paired or unpaired, and one-way, two-way, or three-way analysis of variance (ANOVA). Following significant ANOVAs, a post-hoc test was performed according to the coefficient of variation (CV): Tukey (CV ≤ 15%), Student’s Newman-Keuls (CV 15–30%), and Duncan (CV > 30%). Non-parametric tests were applied to data that did not show normal distribution, using Mann-Whitney, Kruskal – Wallis post-hoc tests, or Wilcoxon matched-pairs signed rank test. Log-rank (Mantel – Cox) test was used for survival analysis. Categorical data were analyzed using Fisher’s exact test, chi-square test, or Wald test, depending on the experimental design. Data are presented as mean ± standard deviation (SD).

## Supplementary Material

Supplemental Material

Supplemental Material

## Data Availability

The 16S rRNA gene libraries produced in this study were deposited in the NCBI SRA database under the project number PRJNA843134. The data are available upon request from the corresponding author.
