# Supplementary material for: Gut microbiota from patients with COVID-19 cause alterations in mice that resemble post-COVID symptoms
Source: Gut Microbes. 2023 Sep 5;15(2):2249146. doi: 10.1080/19490976.2023.2249146 (PMC10481883; doi:10.1080/19490976.2023.2249146)
Supplement: Supplemental Material [file KGMI_A_2249146_SM2945.docx]

Supplementary Materials

**Gut microbiota from patients with COVID-19 cause alterations in mice that resemble post-COVID symptoms.**

Viviani Mendes de Almeida^1*^, Daiane F. Engel^2*^, Mayra F. Ricci^1*^, Clênio Silva Cruz^1^, Ícaro Santos Lopes^3^, Daniele Almeida Alves^4^, Mirna d’ Auriol^5^, João Magalhães^1^, Elayne C. Machado^1^, Victor M. Rocha^1^, Toniana G. Carvalho^7^, Larisse de S. B. Lacerda^8^, Jordane C. Pimenta^8^, Mariana Aganetti^1^, Giuliana S. Zuccoli^6^, Bradley J. Smith^6^, Victor C. Carregari^6^, Erika da Silva Rosa^1^, Izabela Galvão^1^, Geovanni Dantas Cassali^9^, Cristiana C. Garcia^10^, Mauro Martins Teixeira^8^, Leiliane C. André^5^, Fabiola Mara Ribeiro^7^, Flaviano S. Martins^11^, Rafael Simone Saia^12^, Vivian Vasconcelos Costa^8^, Daniel Martins-de-Souza^6,13,14,15^, Philip M. Hansbro^16^, João Trindade Marques^4,17^, Eric R. G. R. Aguiar^3^, Angélica

T. Vieira^1#^


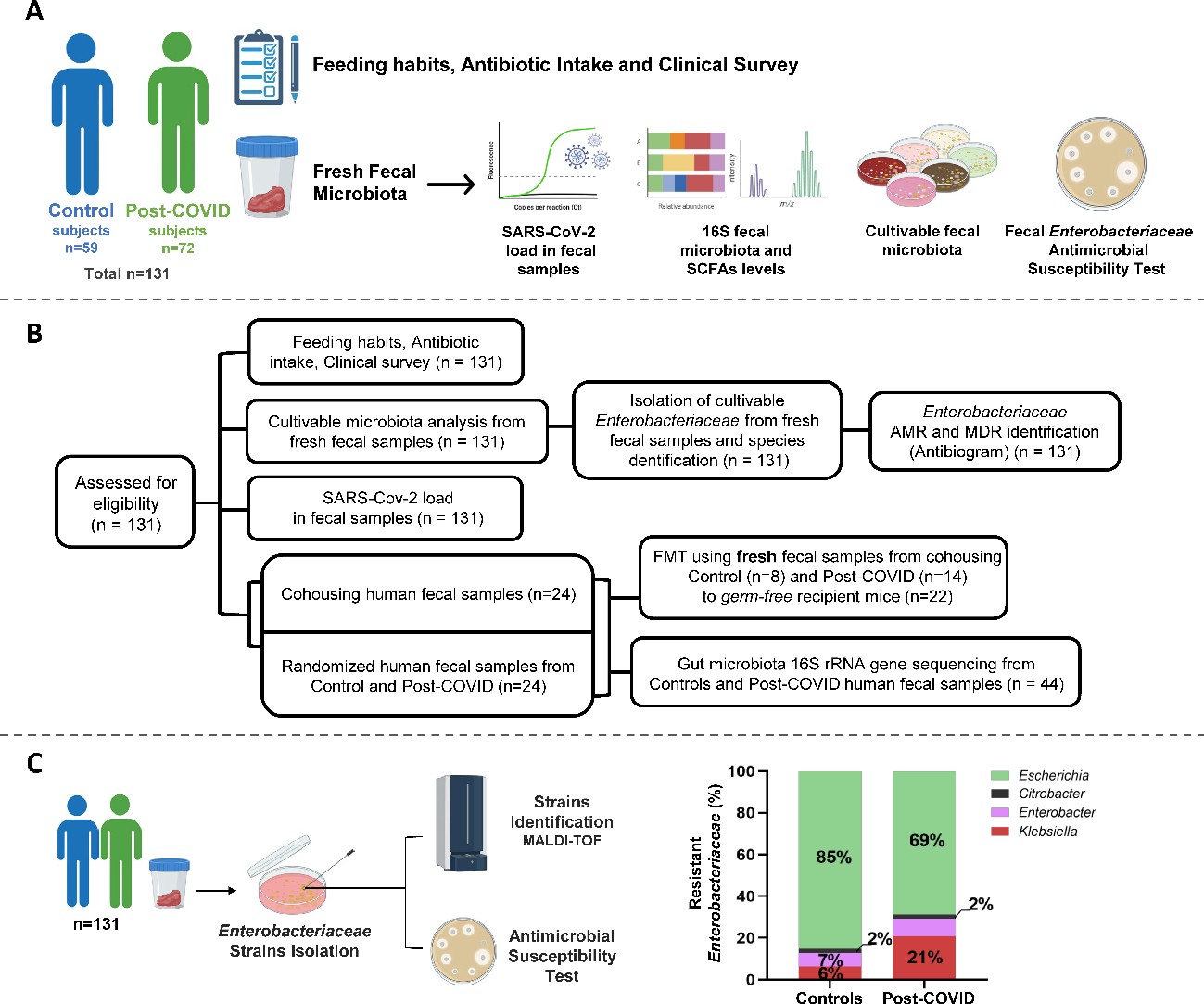


**Figure S1 (related to Figure 1).** Workflow and flowchart for the collection and analysis of post-COVID and control human samples, and analysis of resistant *Enterobacteriaceae* species in human fecal samples. (**A**) Analysis of feeding habits sociodemographic, antibiotics use (at least 4 months before the application of sample collection), and clinical parameters of 59 control and 72 post-COVID subjects. Fresh feces were collected and subjected to SARS-CoV-2 quantification by RT-qPCR, 16S rRNA sequencing, SCFA’s measurements, cultivating fecal microbiota, and antimicrobial susceptibility tests. (**B**) Flowchart of the human samples. (**C**) Descriptive analysis of percentage of resistant *Enterobacteriaceae* species (N=131).


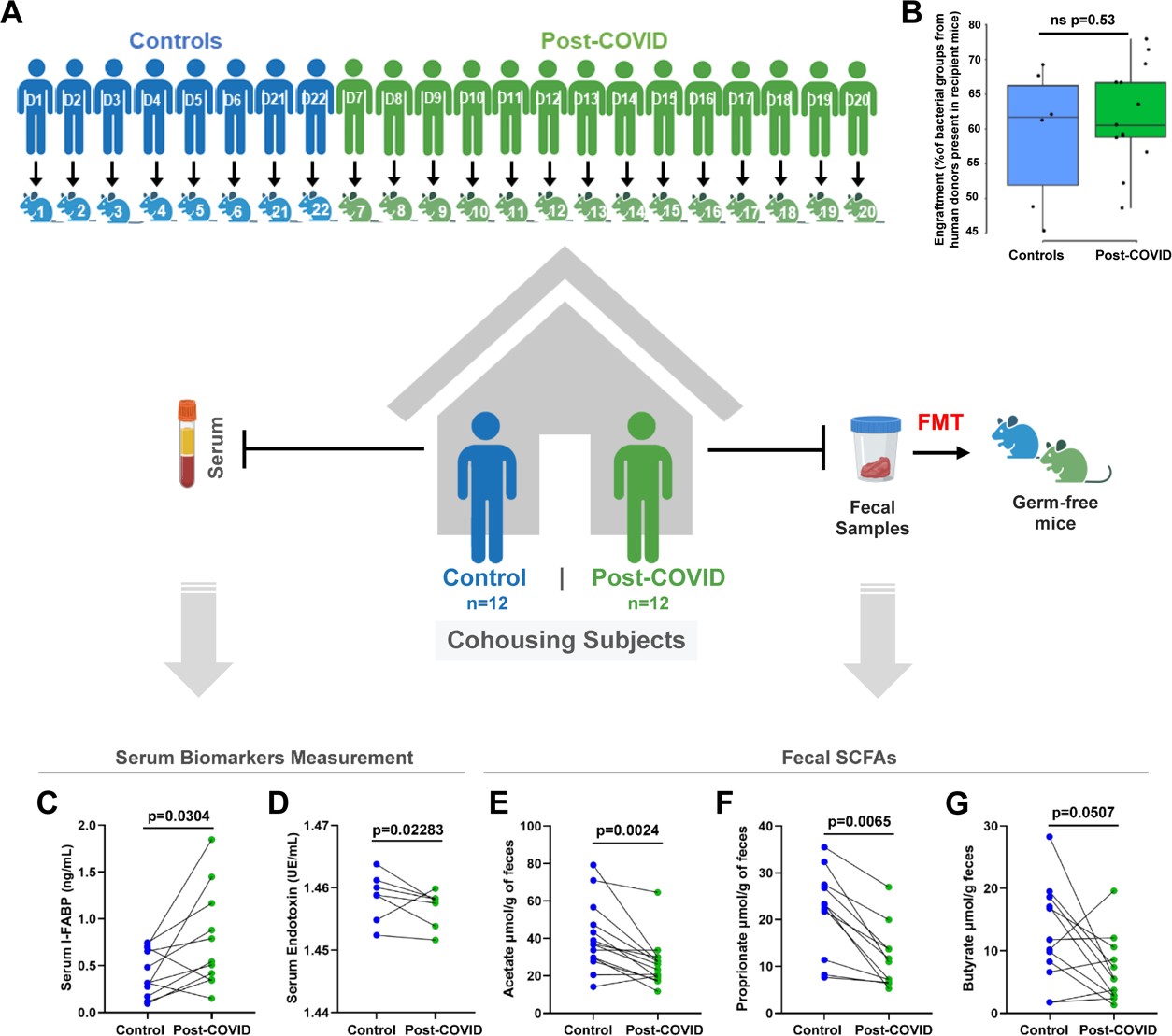


**Figure S1 (related to Figure 2).** Altered in the intestinal homeostasis of post COVID subjects compared to controls from the same household. (**A**) The FMT workflow of control and post-COVID donors performed individually for germ-free mice. **(B)** Prevalence of fecal microbiota engraftment of donor-receptor by bacterial group. Percentage of colonization of computed based on the bacterial group diversity observed in the receptor compared to the diversity observed in the donor. Statistical significance was calculated with the Wilcoxon test. (Human: N=19; Mice: N=22) **(C)** Quantification of serum endotoxin (LPS) levels (N=14). (**D**) Serum I-FABP levels (N=24). Fecal (**E**) acetate, (**F**) propionate, and (**G**) butyrate levels in cohousing post-COVID and controls subjects (N= 24). Statistical analysis: Wilcoxon test was used in B and paired Student’s- t test was used in C-G. Data are shown as mean and standard deviation (SD).


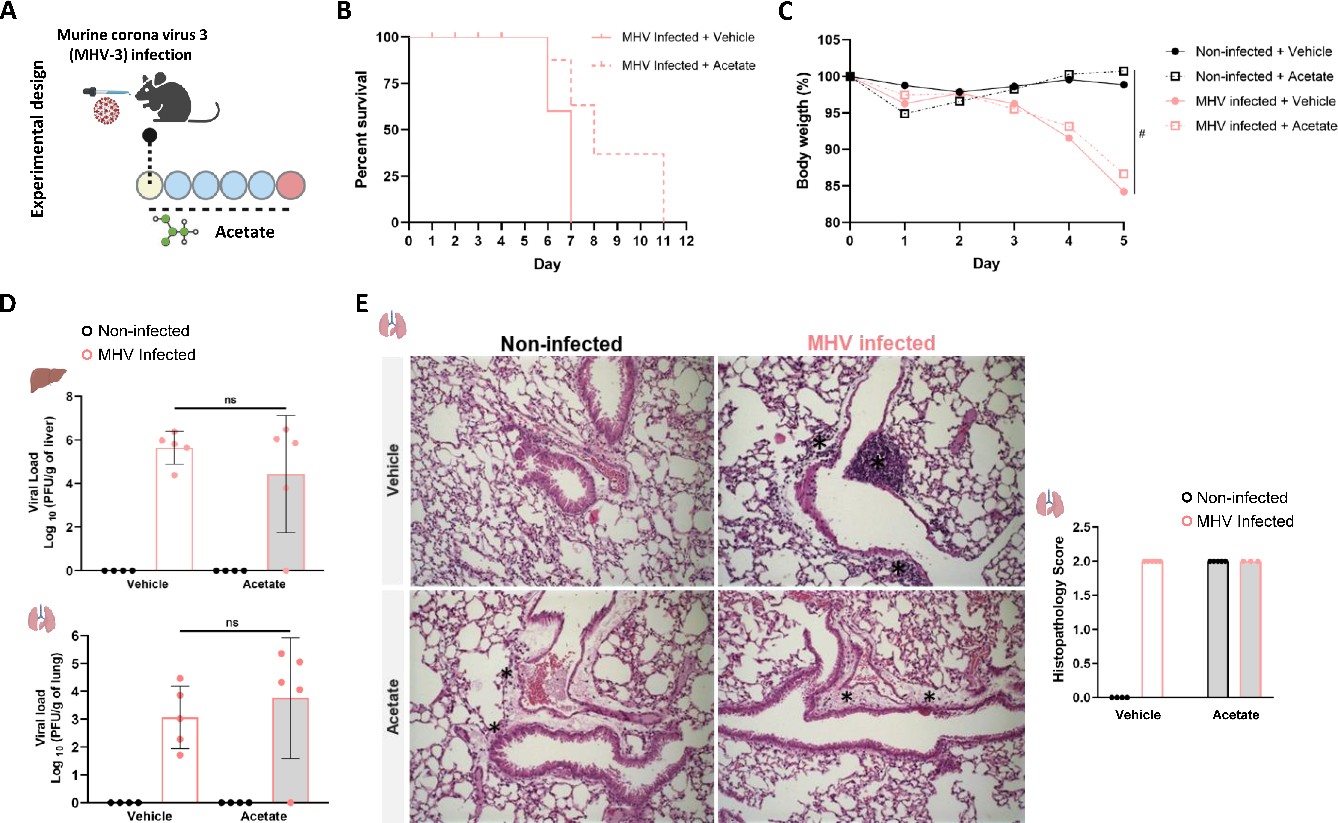


**Figure S1 (related to Figure 6).** The treatment with acetate did not reverse the pulmonary changes observed in mice infected with MHV-3. (**A**) Experimental design: C57BL/6 non-infected and MHV-3 infected and treated with acetate (Vehicle: non- infected N=4; MHV-3 infected N=5; Acetate: non-infected N=4; MHV-3 infected N=5). (**B**) Survival curves evaluated until death of all animals p=0,0013 (N=22) (**C**) Body mass over time measured daily throughout the experiment. (# significant main effect of MHV infection) (N=18). (**D**) MHV-3 titers quantified in the liver and lung extracts by plaque assay (N=18). (**D**) H&E staining: histological alterations in the lung of MHV-3 infected mice and treated with acetate. Graph showing the histopathological score of the airway, vascular and parenchymal inflammation in control and post-COVID mice lungs (N=18). Asterisks indicate inflammatory infiltrates. Scale bar: 50μm. 20X objective. Statistical analysis: log-rank (Mantel–Cox) test was used in B. Three-way repeated measures ANOVA with Tukey’s tests was used in C. Two-way ANOVA with Tukey’s test was used in C, D. and E Data are shown as mean and standard deviation (SD).
